# Percutaneous Resolution of Lumbar Facet Joint Cysts as an Alternative Treatment to Surgery: A Meta-Analysis

**DOI:** 10.1371/journal.pone.0111695

**Published:** 2014-11-12

**Authors:** Feng Shuang, Shu-Xun Hou, Jia-Liang Zhu, Dong-Feng Ren, Zheng Cao, Jia-Guang Tang

**Affiliations:** 1 Department of Orthopaedics, The First Affiliated Hospital of General Hospital of Chinese PLA, Beijing, China; 2 Department of Orthopedics, The 94th Hospital of Chinese PLA, Nanchang, China; The James Cook University Hospital, United Kingdom

## Abstract

**Purpose:**

A comprehensive review of the literature in order to analyze data about the success rate of percutaneous resolution of the lumbar facet joint cysts as a conservative management strategy.

**Methods:**

A systematic search for relevant articles published during 1980 to May 2014 was performed in several electronic databases by using the specific MeSH terms and keywords. Most relevant data was captured and pooled for the meta-analysis to achieve overall effect size of treatment along with 95% confidence intervals.

**Results:**

29 studies were included in the meta-analysis. Follow-up duration as mean ± sd (range) was 16±10.2 (5 days to 5.7 years). Overall the satisfactory results (after short- or long-term follow-up) were achieved in 55.8 [49.5, 62.08] % (pooled mean and 95% CI) of the 544 patients subjected to percutaneous lumbar facet joint cyst resolution procedures. 38.67 [33.3, 43.95] % of this population underwent surgery subsequently to achieve durable relief. There existed no linear relationship between the increasing average duration of follow-up period of individual studies and percent satisfaction from the percutaneous resolutions procedure.

**Conclusion:**

Results shows that the percutaneous cyst resolution procedures have potential to be an alternative to surgical interventions but identification of suitable subjects requires further research.

## Introduction

Facet joint cysts of lumbar spine (LFJCs) are benign degenerative outgrowths which are most usually associated with low back pain and radiculopathy. Two types of cysts recognized under this category are the synovial cysts and ganglion cysts [1]. The synovial cysts have vascularized lining filled with xanthochromic fluid and have communication with facet joint while the ganglion cysts are covered by fibrocartilagenous capsule filled with proteinaceous and gelatinous material and do not communicate with joint [2].

These cysts can arise because of the chronic hypermobility of the spinal segments leading to increased and more frequent loading of the zygapophyseal joint (Z-joint; a synovial joint). This causes the accumulation of fibrocartilaginous substances which provide raw material for cyst formation [3], [4]. The Z-joint is thought to be involved in the genesis of cysts owing to a degenerative process, not fully understood, though herniation of synovial tissue is frequently perceived [5]–[7]. The LFJCs are associated with spinal stenosis, nerve root compression, neurogenic claudication and many other neurological disturbances by encroaching the local foramen [8], [9].

Although, small scale studies indicate that the prevalence of LFJCs in symptomatic patients is 0.7 to 2.5% (Ayberg et al., 2008) [10], but it may be higher and even increase with increasing longevity. This neuropathological agent is strongly associated with late decades of life and females harbor more than males [1].

Diagnosis of the LFJCs utilize magnetic resonance imaging (MRI) or computed tomography (CT) and to some extent CT myelography. Seldom these cysts resolve spontaneously; mostly require treatment. Various management strategies include bed rest, non-steroidal anti-inflammatory drugs, analgesics, physical therapies, transcutaneous electrical nerve stimulation (TENS), intra-articular steroid injections/epidural steroid instillation with or without cyst rupture and CT or flouroscopy guided aspiration of the cyst materials and surgical interventions such as laminectomy, facetectomy, flavectomy, cyst excision and microsurgery.

Long term relief from the symptoms associated with the LFJCs can be achieved with surgery or percutaneous resolution procedures, however. Surgery is the most effective treatment noted so far but studies indicate that percutaneous cyst resolution procedures can be an alternative to surgery in a well-sized subgroup of patients. Moreover, older and high risk patients who are abstained from surgical interventions due to many reasons can also be benefited from later treatment regimen. In order to explore this avenue, this systematic review and meta-analysis is conducted to evaluate the success rate of percutaneous cyst resolution procedures in terms of durable relief and to attempt the identification of subgroup of patients in which chances success with this technique can be better than surgical intervention.

## Materials and Methods

### Study Identification

Detailed systematic search was made in several electronic databases including PubMed/Medline, Embase, EBSCO, CINAHL, Ovid SP, SCI Web of Science and Google Scholar under most relevant keywords. MeSH terms and keywords used in various logical combinations included: spinal, lumbar, cyst, synovial, ganglion, juxtafacet, facet, zygapophyseal, magnetic resonance imaging (MRI), computed tomography (CT), conservative management, percutaneous, puncture, rupture, steroid, injection, intra-articular, epidural, facet, joint, effusion. Literature search was restricted to a period from 1980 to May 2014. All retrospective analyses, prospective studies, and individual case reports were taken into consideration.

### Selection criteria

The PRISMA guidelines were followed for this study. Because of the scarcity of well-designed clinical trials, selection of studies was made under a broader scope and all studies with prospective or retrospective designs and case reports were included. Inclusion criteria were: a) Studies mentioning percutaneous resolution procedures of LFJCs (synovial/ganglion) such as steroid injections, cyst rupture and cyst material aspiration by utilizing CT/fluoroscopically guided instrumentation; b) studies mentioning a short-term or long-term follow-up of the outcomes and related details, including the provision of data of the subjects who underwent surgical procedures in case of failure of the interventions. Exclusion criteria were: a) studies/case reports intervening other types of similar spinal cyst pathologies such as discal cysts, vertebroplasty etc; b) studies involving percutaneous procedures for the purpose of diagnosis only; and c) studies/case reports utilizing percutaneous procedures for the alleviation of back pain without a diagnosis of LFJC/s; d) studies/case reports which did not contain sufficient details of the outcomes of interventions of interest.

### Data extraction, synthesis and analysis

Data were extracted from each research article/case report regarding the demographics of patients, clinical and pathological characteristics, diagnostic tools, procedural features, follow-up period, and outcomes. Outcome measures were the percent satisfactory response of the patient after a reasonable follow-up and the percentage of patients who subsequently underwent surgery. Pooling of dichotomous data (satisfactory outcomes vs surgery requirement) was made by calculating standard errors and 95% confidence intervals (CI) of the data from individual studies and then overall effect size of the meta-analysis was calculated. Forest graphs were plotted manually on the spreadsheets from pooled data and the overall effect size. Descriptive data are presented as mean along with either standard deviation (sd) or range. Quality of the included studies was assessed by using Quality Assessment Tool for Observational Cohort and Cross-Sectional Studies [11].

## Results

**Figure 1:**
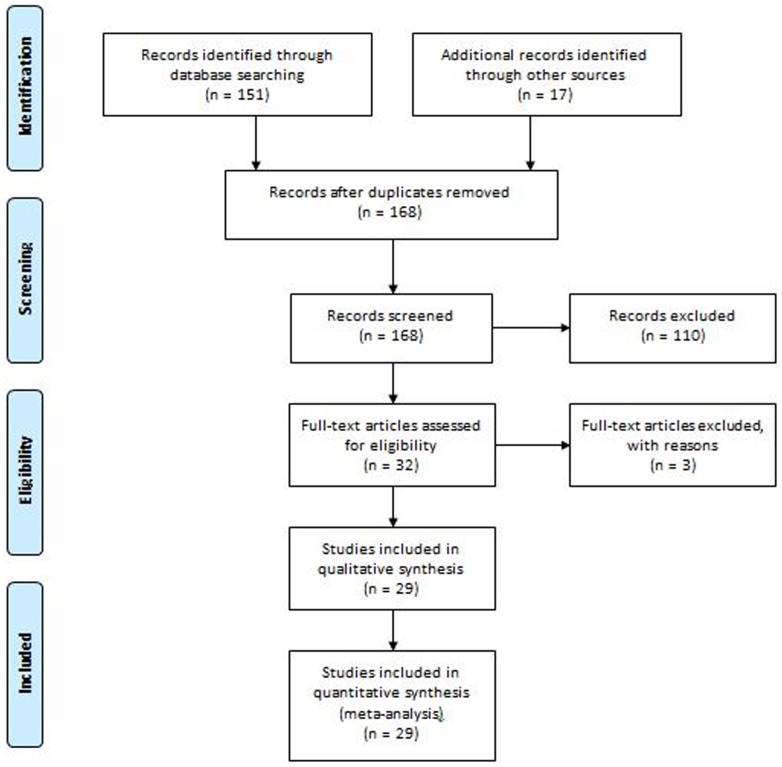
Flowchart of study screening and selection process.

Search identified 29 articles [12]–[40] reporting 12 retrospective studies, 2 prospective studies and 15 case reports which are included in this analytical review. Study screening and selection process has been depicted in Figure 1. Quality assessment outcomes are presented in Table 1.

**Table 1:** Quality Assessment Tool for Observational Cohort and Cross-Sectional Studies.

Criteria	12	13	14	15	16	17	18	19	20	21	22	23	24	25
1. Was the research question or objective in this paper clearly stated?	Y	Y	Y	Y	Y	Y	Y	Y	Y	Y	Y	Y	Y	Y
2. Was the study population clearly specified and defined?	Y	Y	Y	Y	Y	Y	Y	Y	Y	Y	Y	Y	Y	Y
3. Was the participation rate of eligible persons at least 50%?	Y	Y	Y	Y	Y	Y	Y	Y	Y	Y	Y	Y	Y	Y
4. Were all the subjects selected or recruited from the same or similar populations (including the same time period)? Were inclusion and exclusion criteria for being in the study prespecified and applied uniformly to all participants?	Y	Y	Y	Y	Y	Y	Y	Y	Y	Y	Y	Y	Y	Y
5. Was a sample size justification, power description, or variance and effect estimates provided?	N	N	N	N	N	N	Y	N	N	N	N	Y	N	N
6. For the analyses in this paper, were the exposure(s) of interest measured prior to the outcome(s) being measured?	NA	NA	NA	NA	NA	NA	NA	NA	NA	NA	NA	NA	NA	NA
7. Was the timeframe sufficient so that one could reasonably expect to see an association between exposure and outcome if it existed?	N	N	N	Y	Y	N	Y	N	Y	N	N	N	N	N
8. For exposures that can vary in amount or level, did the study examine different levels of the exposure as related to the outcome (e.g., categories of exposure, or exposure measured as continuous variable)?	Y	Y	NA	Y	Y	NA	Y	Y	Y	Y	Y	Y	Y	Y
9. Were the exposure measures (independent variables) clearly defined, valid, reliable, and implemented consistently across all study participants?	Y	Y	Y	Y	Y	Y	Y	Y	Y	Y	Y	Y	Y	Y
10. Was the exposure(s) assessed more than once over time?	Y	Y	Y	Y	Y	NR	Y	Y	Y	Y	Y	NR	Y	NR
11. Were the outcome measures (dependent variables) clearly defined, valid, reliable, and implemented consistently across all study participants?	N	N	N	Y	Y	N	Y	N	Y	N	N	N	N	N
12. Were the outcome assessors blinded to the exposure status of participants?	N	N	N	N	N	N	N	N	N	N	N	N	N	N
13. Was loss to follow-up after baseline 20% or less?	CD	CD	CD	CD	CD	CD	CD	CD	CD	CD	CD	CD	CD	CD
14. Were key potential confounding variables measured and adjusted statistically for their impact on the relationship between exposure(s) and outcome(s)?	N	N	N	N	Y	N	Y	N	N	N	N	N	N	N

Legends: CD: Cannot be determined, NA: not applicable, NR: not reported, N: no, Y: yes.

**Figure 2:**
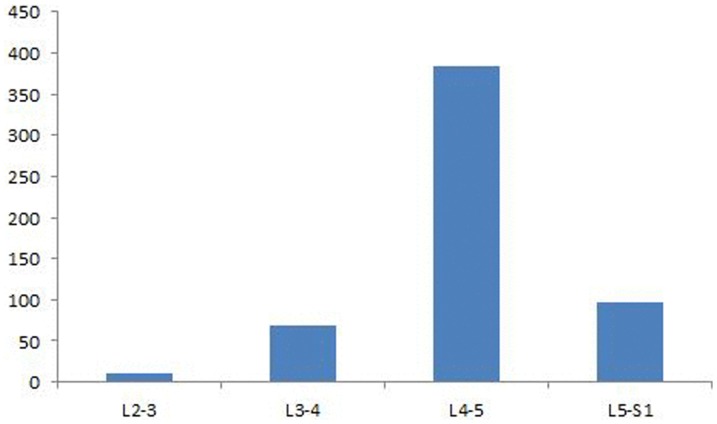
Spinal level of cysts diagnosed in the patients included in the meta-analysis.

Major characteristics relevant to the manifesto of the present study are present in Table 2. Overall of the 544 subjects included in this meta-analysis, age of the participants as mean ± sd (range) was 62±4.2 (28–87) years and proportion of females in this population was 64%. Spinal level of the cysts was L_2–3_ in 10, L_3–4_ in 69, L_4–5_ in 384 and L_5_–S_1_ in 96 cases (Figure 2). Size of the cyst ranged from 6×13 to 12×18 mm. Duration of symptoms before percutaneous resolution interventions ranged from 2 weeks to 60 months. Major conditions associated with the presence of LFJCs in these patients were lower back pain and radiculopathy, especially lower extremity radiculopathy. Symptomatic features at clinical presentation are presented in Table 3.

**Table 2:** Characteristics of the included studies which utilized percutaneous resolution of lumber facet joint cyst procedures.

Study/Design	Patients' characteristics	Pathology	Diagnosis	Intervention	Follow up	Outcome
Allen et al., 2009 [12]/Retrospective cohort	n: 32; age: 66 (46–86) y; females: 18; Location (L_3–4_/L_4–5_/L_5_–S_1_): 2/22/8 (left 18, right 13, bilateral 1)	LBP/LER since 5 mo	MRI	FCR/ESI	12 (6–24) mo	Satisfactory: 19 (60%), Repeats: 11 (34%), Required Surgery: 6 (19%)
Amoretti et al., 2012 [13]/prospective	n: 120; age: 68.2 (52–84) y; Location (L_3–4_/L_4–5_/L_5_–S_1_): 16/84/20; VAS change; mean ± sd: 7.2±1.2 to 2.9±1.2	Disabling LBP/radiculopathy	MRI	CTISI	12 mo	Satisfactory: 90 (75%), Repeats: 43 (36%), Required Surgery: 30 (25%)
Bjorkengren et al., 1987 [14]/prospective	n: 3; age: 59 (44, 56 & 77) y; females: 2; Location: L_4–5_ in all	LBP/LER	CT	CTISI	11 (6–14) mo	Satisfactory: 2 partially, Repeats: 1, Required surgery: 1/refused
Bureau et al., 2001 [17]/retrospective	n: 12; age: 60 (45–79) y; females: 8; Location (L_3–4_/L_4–5_/L_5_–S_1_): 1/10/1; Cyst size: 11×13.6 (6–13×8–19)mm	LBP/radiculopathy	MRI	FCR/SI	23 (12–36) mo	Satisfactory: 9 (75%), Repeats: 7 (58%), Required Surgery: 3 (25%)
Cambron et al., 2013 [18]/retrospective	n: 110; age: 63 (28–87) y; females: 71; Location (L_2–3_/L_3–4_/L_4–5_/L_5_–S_1_): 6/17/89/22; Cyst size: 10.6 mm/intensity: H 48/L 65	LER	MRI	CT-guided FCR/SI	34 (7–93) mo	Satisfactory: 63 (57%), Repeats: 40 (36%), Required Surgery: 47 (43%)
Carrera, 1980 [19]/retrospective	n: 20; age (mean): 54 y; females: 12; Location (L_2–3_/L_3–4_/L_4–5_/L_5_–S_1_): NA	LBP/symptomatic facet arthropathy	CT	IAFB	6–12 mo	Satisfactory: 6 (30%), Repeats: NA, Required Surgery: NA
Martha et al., 2009 [30]/retrospective	n: 101; age: 59.8±1.3 y; females: 69; Location (L_2–3_/L_3–4_/L_4–5_/L_5_–S_1_): 2/9/69/21	LBP/LER	MRI	FCR/SI	3.2±1.3 y (mean ± sd)	Satisfactory: 46 (46%), Repeats: 51 (51%), Required Surgery: 55 (55%)
Ortiz & Tekchandani, 2013 [32]/retrospective	n: 20; age: 65.5 y average; females: 9; Location (L_2–3_/L_3–4_/L_4–5_/L_5_–S_1_): 1/5/11/4; Cyst size: 7.3 (3–14) mm	LBP/LER	NA	CTISI/aspiration	18 (4–24)	Satisfactory: 18 (90%), Repeats: 4 (20%), Required Surgery: 2 (10%)
Parlier-Cuau et al., 1999 [33]/retrospective	n: 30; age: 67 (44–82) y; females: 21; Location (L_2–3_/L_3–4_/L_4–5_/L_5_–S_1_): 1/3/25/1; Symptom duration: at least 6 mo	Sciatic/femoral pain	CT: 27/MRI: 3/arthrography	FISI	26 (8–50) mo	Satisfactory: 14 (47%), Repeats: 7 (23%), Required Surgery: 14 (47%)
Sabers et al., 2005 [35]/retrospective	n: 23; age: 64 (28–81) y; females: 12; Location (L_3–4_/L_4–5_/L_5_–S_1_): 1/15/7; Symptom duration: 10.5 (2 wk–48 mo)	LBP/LER	MRI	FISI/aspiration	9.1 (1.5–21) mo	Satisfactory: 9 (50%), Repeats: 2 (1–4) per subject, Required Surgery: 9 (50%)
Sauvage et al., 2000 [36]/retrospective	n: 13; age: 63 (42–87) y; females: 9; Location (L_3–4_/L_4–5_/L_5_–S_1_): 1/8/4; Cyst size: 9 (5–11) mm; largest 12×18 mm	radiculopathy	MRI	CTISI	9 (2–25) mo	Satisfactory: 6 (46%), Repeats: 6 (46%), Required Surgery: 3 (23%)
Schulz et al 2011 [37]/prospective	n: 20; age: median 54.5 y; females: 17; Location (L_3–4_/L_4–5_/L_5_–S_1_): 1/19/0; Symptom duration: median 10.5 mo	radiculopathy	CT	CTISI	24 mo	Satisfactory: 8 (40%), Repeats: NA, Required Surgery: 12 (60%)
Shah and Lutz, 2003 [38]/retrospective	n: 10; age: 60 (53–70) y; females: 8; Location (L_3–4_/L_4–5_/L_5_–S_1_): 0/8/2; Symptom duration: 7.9 (1–30) mo	LBP/LER	CT/MRI	FISI/aspiration/ESI	11.5 (3–30) mo	Satisfactory: 1 (10%), Repeats: 1 (10%), Required Surgery: 8 (80%)
Slipman et al., 2000 [40]/retrospective	n: 14; age: 60.2 (39–87) y; females: 7; Location (L_3–4_/L_4–5_/L_5_–S_1_): 2/10/2; Symptom duration: 18.8 (3–60) mo	radiculopathy	CT/MRI	FISI/aspiration	1.4 (1–3) y	Satisfactory: 4 (40%), Repeats: NA, Required Surgery: 8(58%)
Case Reports						
Boissiere et al, 2013 [15]	57 y old male with cyst at L_4–5_	Sciatica since 24 mo	CT	CTISI	6 mo	surgery (decompression + fusion)
Braza et al., 2005 [16]	48 y old man with cyst at L_4–5_ (7 mm)	Thigh and calf pain (7 mo)	MRI	FISI/aspiration	2 mo	80% improvement in pain relief
Casselman et al 1985 [20]	65 y old woman with cyst at L_4–5_	LBP/LER	CT	Intra-articular SI	3 mo	Underwent surgery
Chang et al 2009 [21]	63 y old woman with cyst at L_5_–S_1_ (7 mm)	Left-sided radiculopathy	MRI	CT-guided FISI	1 mo	Satisfactory relief
Foley, 2009 [22]	44 y old man with cyst at L_4–5_	LBP	MRI	FISI/rupture	1 mo	Satisfactory relief (0/10 VAS)
Gishen & Mill., 2001 [23]	65 y old woman with cyst at L_5_–S_1_	Hip osteoarthris/left sciatica	MRI	CTISI/ESI	12 mo	Satisfactory (asymptomatic)
Hong et al., 1995 [24]	51 y old woman with cyst at L_4–5_	LBP/right knee pain (6 mo)	MRI	FCA, no SI	6 mo	Satisfactory (asymptomatic)
Imai et al., 1998 [25]	77/55 y old women with cysts at L_4–5_/L_3–4_	LBP/LER (15 mo/10 mo)	MRI	FISI/aspiration	5 d/2 mo	surgery for durable relief (both)
Kozar & Jer. 2014 [26]	77 y old man with cyst at L_4–5_ (3×5 mm)	LBP/LER (3 y)	MRI	CTISI/rupture	1 mo	Partial relief/surgery not feasible
Lim et al., 2001 [27]	67 y old woman with cyst at L_4–5_	LBP/right LER	MRI	CTISI	9 mo	Satisfactory (asymptomatic)
Lin et al., 2014 [28]	52 y old man with cyst at L_4–5_	LBP/right LER since 10 mo	MRI	UISI	18 mo	Satisfactory (asymptomatic)
Lutz and Shen, 2002 [29]	48 y old woman; cyst at L_4–5_ (7×15 mm)	LBP/right LER (4 mo)	MRI	FCA, no SI	1 mo	Satisfactory (asymptomatic)
Melfi & Aprill, 2005 [31]	72 y old woman with cyst at L_4–5_/L_5_–S_1_	Chronic LBP/LER (7 mo)	MRI	FISI	30 mo	Satisfactory (asymptomatic)
Rauchwerger 2011 [34]	70 y old woman with cyst at L_5_–S_1_	LBP/radiculopathy (1 y)	MRI	FISI	1 day	Partial relief
Shin et al., 2012 [39]	51 y old man with cyst at L_4–5_	LBP/LER (1mo)	MRI	FISI/aspiration	6 mo	Satisfactory (asymptomatic)

Values are presented as mean (range) unless otherwise stated. Abbreviations: CTISI (CT-guided Intra-cystic/Intra-articular SI), ESI (epidural SI), FCA (fluoroscopically-guided cyst aspiration), FCR (fluoroscopically guided cyst rupture), FISI (fluoroscopic intra-articular SI), IAFB (intra-articular facet block), LBP (lower back pain), LER (lower extremity radiculopathy), mo (month/s), NA (not available), SI (steroid injection), wk (week/s), y (year/s).

**Table 3:** Common presenting conditions of lumbar facet joint cysts.

Low back pain	Disc herniation
Unilateral or bilateral radiculopathy	Spinal stenosis
Myelopathy	Neural foraminal stenosis
Neurogenic claudication	Herniated nucleus pulposus
Caudaequina syndrome	Osteoarthritis
Intracystic or epidural hemorrhage	Arachnoiditis
Spondylolisthesis	Cauda equina compression from cyst
Trochanteric bursitis	High-intensity zone in disk
Peripheral neuropathy	

The procedures involved cyst puncture, rupture, aspiration, intra-articular steroid injection, epidural steroid injection, and local anesthetics injections. These procedures were performed under CT/fluoroscopic guidance, though, not all studies utilized each of these interventions. Arthrography was also performed in majority of cases. Majority of the subjects were diagnosed with MRI (about 85% vs CT about 15%) for harboring one or more LFJCs. Cyst rupture outcomes were assessed by the loss of resistance method or by the extravasation of dye.

**Figure 3:**
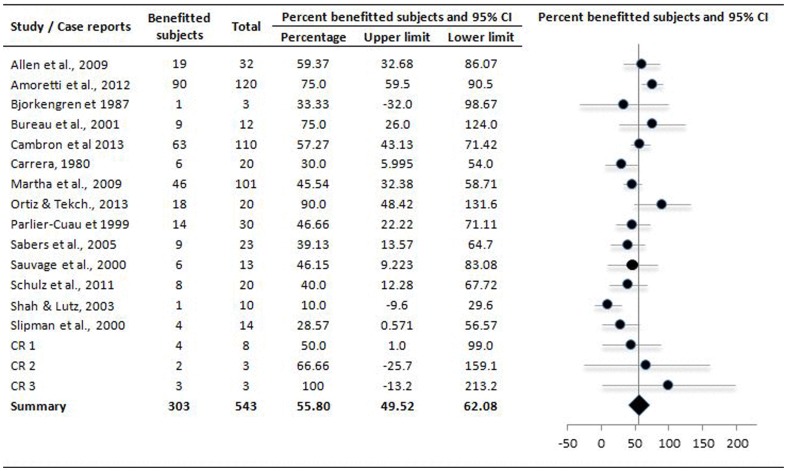
Forest plot showing effect sizes of satisfactory results of percutaneous treatments of the LFJCs after short- or long-term follow-up in individual studies (closed circles) and the overall effect size achieved in meta-analysis (diamond). CR 1 (follow-up 1 mo): Braza et al., 2005; Casselman et al., 1985; Chang, 2009; Foley, 2009; Imai et al., 1998; Kozar & Jeromal, 2014; Lutz and Shen, 2002; Rauchwerger et al., 2011/CR 2 (follow-up 6 month): Boissier et al., 2013; Hong et al., 1995; Shin et al., 2012/CR 3 (follow-up 1 year or more): Gishen et al., 2001; Lim et al., 2001; Lin et al., 2014; Melfi and Aprill, 2005.

**Figure 4:**
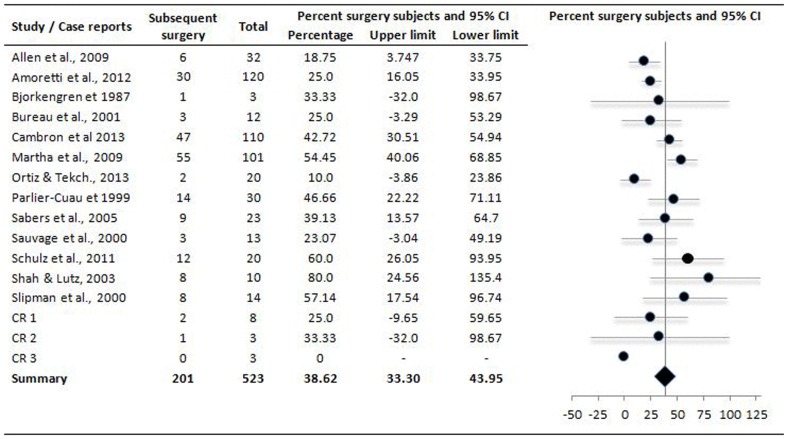
Forest plot showing effect sizes of subjects underwent surgical treatments subsequent to failure of percutaneous treatments of the LFJCs in individual studies (closed circles) and the overall effect size achieved in meta-analysis (diamond). CR 1/CR 2/CR 3 as given in Figure 2.

Follow-up duration as mean ± sd (range) was 16±10.2 (5 days to 5.7 years). Overall the satisfactory results (after short- or long-term follow-up) were achieved in 55.8 [49.5, 62.08] % (pooled mean and 95% CI) of the 544 patients subjected to percutaneous lumbar cyst resolution procedures (Figure 3). Repeat procedures were performed in 115 of 323 subjects at an average duration of 4.7 (range 0.06–26.3) months after first procedure (data from 7 studies only). On the other hand, 38.67 [33.3, 43.95] % of this population underwent surgery subsequently to achieve durable relief (Figure 4). Average time from percutaneous resolution procedure to surgery was 6.7 (range 0.13–34.4) months (data from six studies only).

**Figure 5:**
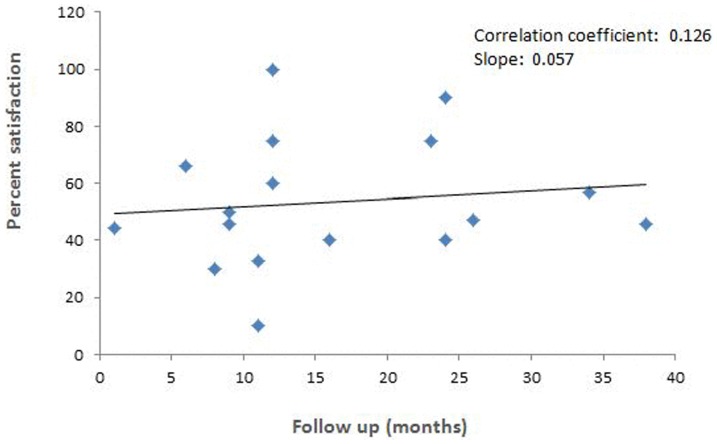
Scatter plot showing relationship between percent satisfaction of the subjects of percutaneous resolution procedures and follow-up duration in months.

There was no purposeful linear relationship between the increasing average duration of follow-up period of individual studies and percent satisfaction from the percutaneous resolutions procedure (correlation coefficient: 0.13; slope: 0.057; Figure 5). However, number of studies with around 1-year follow up was highest (10), with 2-year follow-up 4 and with 3-year follow-up 2 only. For this analysis individual case reports were lumped in to three groups according to follow-up period (1, 6 and 12 months). Only one case report had a follow-up of over 2-years duration (30 months).

## Discussion

Usually, the LFJCs are found as rare incidental MRI findings of elderly patients (usually in their 6^th^ or 7^th^ decade) presenting with low back pain and lower extremity radiculopathy. However, discovery of LFJCs remains difficult because low back pain is one of the most common presentations in a visit to physician [41]. Frequently, small cohorts of patients often develop additional bony abnormalities, including instability and spondylolisthesis.

Previously, it was difficult to pinpoint a precise existence of a cyst. Rather, the physician relied on his/her clinical acumen. For example, bilateral examinations of L_4_, L_5_ and S_1_, supplying the knee, foot dorsiflexion and plantar flexion, respectively, could give quick insight into the functioning of these spinal nerve roots. Added to these were lumbosacral flexion-extension plain film radiographs that could provide basic information about vertebral anatomy. However, with the advent of modern imaging modalities like CT scans and MRI, primary care physicians as well as specialists started utilizing these techniques in order to obtain more reliable anatomical features leading to pathology. This has resulted in better insights of pathoanatomical diagnoses that can provide sustained and earlier relief.

The present study utilizes almost all relevant data to appraise the success rate of the percutaneous resolution of the LFJCs and finds perhaps the highest rate (56%) reviewed so far [2 e.g.]. This appears to be because of inclusion of 15 case reports which provide considerable power to analysis. Overall success rate noted in the case reports was 70%, whereas, in the pooled analysis of 14 studies the success rate was noted to about 50%. Although, follow-up period of the case reports was much less than the pooled analysis of 14 studies, yet, in the subset of 4 case reports with 9, 12, 18, and 30 months follow-up, the success rate was 100%. Overall association between the follow-up and satisfactory results was also not providing indication of declined success rate with increments in follow up period. Such a difference of success rate of percutaneous procedures in the retrospective analyses and case reports can be attributed to publication bias or scarcity of prospective studies will be clarified in future research. Nevertheless, this point is encouraging enough to provide impetus for larger and longer trial/s to assess the potentials of this treatment strategy.

Natural history of the disease progression of LJFCs is not known. Frequently, patients with radicular pain may be advised for obtaining MRI scans and if there is incidental detection of LJC, detailed neurological examination is meritorious in order to seek insights into the associated pathophysiology. Patients presenting with any kind of radicular pain or associated claudication syndromes, cauda equina syndrome, or any lower extremity motor or sensory symptoms must be evaluated with advanced imaging like MRI. However, in order to avoid extra un-forecasted healthcare costs, there is sheer need of a good clinical examination at the presentation. Due to methodological issues, scarcity of categorical data and statistical power limitations, the present study could not arrive at an initiative of establishing criteria for the selection of suitable patients for percutaneous resolution procedures. Narrowing and ideally eliminating the gray areas of when to take the decision for percutaneous rupture versus the definitive strategy of cyst excision remains the hallmark of clinical research in this area. Surgical excision is precise, but is time consuming, expensive and still not risk-free. On the other hand complications may also develop following procedures such as paraplegia [42].

Because of a number of factors, the present study encounters significant limitations. Firstly, as the diagnosis of LJC remains incidental, there is only one considerable sized prospective study and all others are either retrospective analyses or case reports. Schulz et al. [37] utilized a prospective design to compare the efficacy of percutaneous resolution of LFJCs with microsurgery and noticed a clear-cut supremacy of microsurgery over percutaneous resolution attempts. Their study was not randomized but acts as a required initiative which noted satisfactory benefit of percutaneous treatment for 8 of 20 patients. Indeed, because of minimally invasiveness of this treatment, it remains a treatment of choice.

Secondly, follow-up period in the majority of studies was less than two years which makes it difficult to speculate long-term benefits of the intervention. Thirdly, data availability remained a major issue as it could be useful to apply meta-regression analyses for predicting factor by utilizing data such as age, gender cyst size, cyst type, cyst orientation/location, radiological intensity, pre-procedure duration of symptoms and previous history of treatment/s. Although, case reports were considerably detailed yet in many all relevant data was not available. Cambron et al. [18] studied the effect of low or high signal intensity of MRI on the success rate of percutaneous resolution of LFJCs and noted that patients with T2-hyperintene LFJCs can be more reliably benefited from percutaneous resolution procedures.

It seems that the success rate of percutaneous resolution procedures will increase with the improvement in decision-making information and advancement in technology and skill training and exposure. However, of much importance is the availability of results of a few or a bigger, multi-center randomized controlled trial/s with adequate power to assess the success rate as well as the predicting factors for percutaneous resolution procedure selection. Without which as pointed out by Arnold et al. [43], patient is presented with the coin flip odds for percutaneous vs surgery choice.

## Conclusion

By analyzing all available evidence pertaining to the efficacy of percutaneous cyst resolution procedures the present study finds this therapeutic regimen as an alternative to surgical interventions but is unable to identify subgroup/s of patients that can be benefited more reliably with this technique and therefore urges to conduct comparative studies with longer follow-up periods.

## Supporting Information

Checklist S1 **PRISMA Checklist.**
(PDF)
